# Wnt1 Participates in Inflammation Induced by Lipopolysaccharide Through Upregulating Scavenger Receptor A and NF-kB

**DOI:** 10.1007/s10753-015-0147-8

**Published:** 2015-03-07

**Authors:** Wenting Zhao, Zewei Sun, Shuai Wang, Zhenwei Li, Liangrong Zheng

**Affiliations:** Department of Cardiology, First Affiliated Hospital, College of Medicine, Zhejiang University, 79Qingchun Road, Hangzhou, 310003 China

**Keywords:** LPS, inflammatory factors, wnt1, NF-kB, scavenger receptor A (SRA)

## Abstract

The study investigated the role of wnt1 in the inflammatory response initiated by lipolysaccharide (LPS), and analyzed the association between wnt1, NF-KB, and inflammatory factors. THP-1 cells were activated with phorbol-12-myristate-13-acetate (PMA) and treated with LPS to induce inflammation. THP-1 cells were transfected with *wnt1*siRNA and overexpression plasmid to explore the relationship among wnt1, SRA, and NF-KB. Inhibitor of β-catenin and siRNA of *FZD1*were used to investigate the signaling events involved in SRA activation induced by wnt1. Levels of NF-kB protein and inflammatory cytokines were assessed following*wnt1* siRNA and LPS treatment. PMA activation and LPS treatment of THP-1 cells increased wnt1 protein levels. Wnt1 promoted SRA expression through activation of canonical wnt pathway. Wnt1 increased NF-kB protein levels and enhanced the secretion of IL-6, TNF-α, and iNOS through binding to SRA. These findings suggest that wnt1 increased SRA and NF-kB protein levels and participated in the inflammatory response.

## INTRODUCTION

Lipopolysaccharide (LPS) is released by Gram-negative bacteria and elicits potent immune responses from mammalian cells, including the secretion of pro-inflammatory cytokines. Recognition of LPS by innate immune factors through surface receptors is a key process during septic shock. Both scavenger receptor A (SRA) and toll-like receptor 4(TLR4) are involved in the activation of NF-kB induced by LPS.

SRA, either promoted by GM-CSF or PMA, is a multifunctional receptor that promotes lipid uptake by macrophages [1]. SRA mediates the regulation of the B cell activation state [2] and suppresses CD8 T cell activation by down-regulating TLR4 signaling [3]. Evidence suggests that LPS regulates SRA expression in macrophages. In apoE-deficient mice, which spontaneously develop atherosclerotic lesions on a standard chow diet, SRA was involved in the activation of NF-kB induced by LPS through an interaction with TLR4. LPS exposure enhanced SRA association with TLR4 on the cell surface in presence of fucoidan, a ligand of SRA [4].

Wnt1 is known to activate NF-kB in PC12 cells. The canonical wnt pathway causes an accumulation of β-catenin in the cell cytoplasm and is involved in several disease processes [5–7]. Previous studies have shown that GSK-3β, an inhibitory protein of β-catenin, is required for toxic shock induced by LPS through apoptosis signal-regulating kinase 1(ASK1) [3]. Other results show that wnt1 is involved in the activation of NF-kB in PC12 cells [8]. Our preliminary data demonstrate that wnt1 is produced in THP-1 cells following treatment with phorbol-12-myristate-13-acetate (PMA) or LPS.

Detailed knowledge of the regulation of SRA by LPS and activation of NF-kB by wnt1 is lacking. This study aims to investigate the role of the wnt1 in the inflammatory response initiated by LPS and analyze the association among wnt1, SRA, and NF-KB.

## MATERIALS AND METHODS

### Study Design

Cells from the human monocytic cell line THP-1 were treated with LPS to induce inflammation. THP-1 cells were transfected with *wnt1* siRNA and overexpression plasmid to explore the relationship between wnt1 and SRA. Inhibitors of the canonical wnt pathway and siRNA of FZD1were used to investigate the signaling events involved in SRA activation by wnt1. Changes in levels of NF-kB protein were assessed following siRNA-wnt1 and LPS treatment. Levels of inflammatory cytokines were detected by ELISA and Western blots.

### Cell Culture and Transfection

THP-1 cells were cultured in complete RPMI-1640 medium with 10 % fetal bovine serum (Gibco, USA). Before all experiments, cells were incubated with PMA (100 nmol/ml) (Sigma, USA) for 24 h until confluence to make THP-1 became macrophages-like sticky cells. Subsequently, LPS from *Escherichia coli* O127:B8 (Sigma, USA) was used to induce endotoxemia. Inhibitor of β-catenin (VAX939 50 nmol, Sigma, USA) was added to cells 30 min before LPS treatment or plasmid transfection.

For all transfection experiments, cells were washed with PBS twice and cultured in serum-free medium for 30 min according to the manufacturer’s instructions. For siRNA transfection, cells were incubated with 1 μl siRNA (50umol) (Baiao, China) in 200 μl RPMI-1640 for 5 min, and 3.3 μl RNA iMAX transfection reagent (Lifetech, USA) for 10 min. The suspension was added to a six-well plate. For plasmid transfection, cells were incubated with 1000 ng wnt1 plasmid (OriGene, USA) or 5 μl complementary DNA (cDNA) transfection reagent (Pufei, China) in 100 μl RPMI-1640 for 5 min. Mixtures were combined, incubated for 15 min, and added to a six-well plate. After 4 h, cell medium was replaced with complete medium. All cells were harvested after 24 h for later detection.

### Western Blot

Proteins were purified from cultured cells. Briefly, samples were lysed by boiling for 5 min, and 30 μg total protein was subjected to SDS gel electrophoresis (10 % polyacrylamide). Proteins were blotted onto a polyvinylidene difluoride (PVDF) membrane (Millipore, USA) and incubated at 4 °C overnight with primary antibodies: rabbit anti-iNOS (1:1000 millipore USA), mouse anti-SRA (1:1000 CST, USA), rabbit anti-p65 (1:1000 beyotime, CHINA), mouse anti-wnt1 (1:1000 millipore, USA), or rabbit anti-GAPDH (1:1000 CST, USA). The next day, proteins were incubated for 2 h at room temperature with secondary antibodies: goat anti-mouse or anti-rabbit (1:5000 Kangwei, China). ECL Western Blotting Substrate was used for detection (Pierce, USA).

### Co-immunoprecipitation

Cells were lysed by RIPA lysis buffer (Beyotime, China). After centrifugation, 50 μg of protein lysate were pretreated with 20 μl protein A/G agarose (Santa Cruz, USA) and incubated for 1 h at 4 °C with gentle rotation. Lysates were incubated with 1 μg anti-p65 (Abcam USA) at 4 °C overnight with gentle rotation. The next day, 80 μl protein A/G agarose was added, and the suspensions were incubated for 2 h at 4 °C with gentle rotation. Suspensions were centrifuged, and the agarose phase collected and washed three times with PBS. Samples were eluted with 30 μl protein lysis buffer (Beyotime, China). After elution, loading buffer (7.5 μl of a 5x solution; Beyotime, China) was added, and samples were boiled for 5 min. For Western blotting, anti-wnt1 or anti-SRA antibody (1:1000, CST/millpore, USA) was used to detect the protein.

### Immunofluorescence Analysis

THP-1 cells were cultured in the cell culture dish (NEST, USA). After different treatments, the cells were fixed and permeabilized then incubated with anti-wnt1 or anti-SRA antibody (1:100) at 37 °C for 1 h, after washed with PBS and stained with goat-anti-rabbit rhodamine IgG or goat-anti-mouse FITC IgG (Kangwei, China) (1:100) at 37 °C for 1 h, followed by DAPI staining (guge, CHINA). The cells were examined using a Zeiss Confocal Imaging System (Carl Zeiss, Germany).

### ELISA Assay

Following different treatments, THP-1 cell supernatants were collected after centrifugation for 10 min at 3000 rpm. Inflammatory factors were identified using TNF-α (BD USA), and IL-6 ELISA (R&D USA) kits according to the manufacturer’s instructions.

### Statistical Analysis

Statistical analyses were performed using Graphpad Prism 5.0. Experiments were repeated in triplicates. Data are reported as means ± standard deviations (SD). Between-group differences were assessed using student’s test. A value of *P* ≤ 0.05 was considered statistically significant.

## RESULTS

### Wnt1 Was Induced by PMA Activation and LPS Treatment

**Fig. 1:**
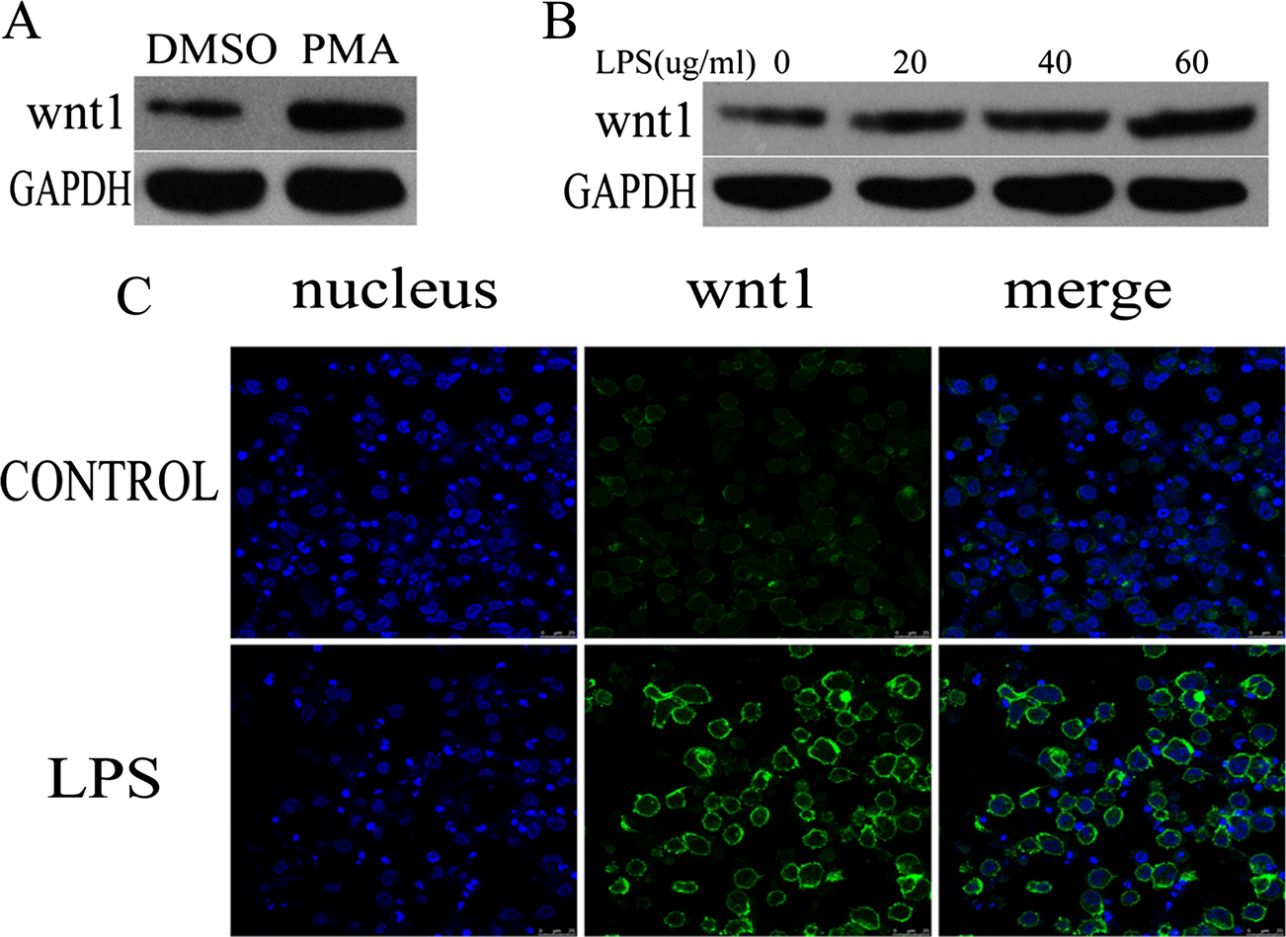
wnt1 was increased in LPS-stimulated THP-1 cells. **a** wnt1 protein level was increased in THP-1 cells treated with PMA (100 nmol/ml for 24 h) (*p* < 0.05). **b** wnt1 protein level was increased in a dose-dependent manner in THP-1 cells treated with LPS (0–60 μg/ml) (*p* < 0.05). **c** THP-1 cells were cultured in the presence or absence of LPS (40ug/ml) for 24 h. Confocal microscopy imaging of wnt1 (*green*) was shown. Results were normalized against levels of GAPDH protein.

Levels of wnt1 protein were increased in PMA-activated THP-1 cells compared to controls (Fig. 1a). The levels of wnt1 was further increased in LPS-stimulated THP-1 cells in a dose-dependent manner (LPS 0–60 μg/ml) (Fig. 1b). Moreover, confocal image systems showed that wnt1 mainly distributed in macrophages surface, and LPS stimulation strengthened it (Fig. 1c).

### NF-KB and Inflammatory Factors Were Activated by wnt1

**Fig. 2:**
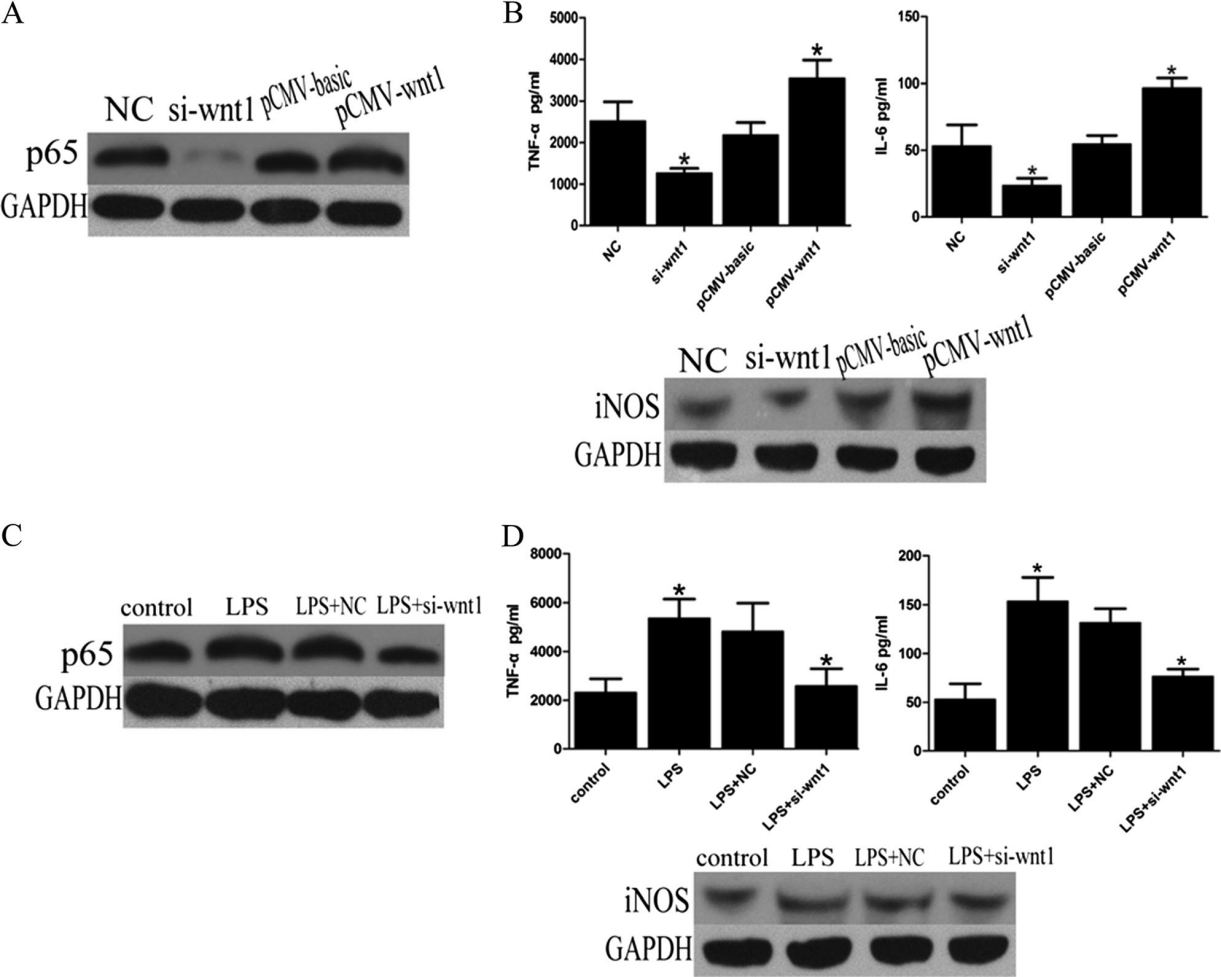
wnt1 promotes secretion of inflammatory factors through stimulating NF-kB pathway. **a** p65 protein level was decreased in THP-1 cells transfected with *wnt1* siRNA while ectopic expression of wnt1 increased it (*p* < 0.05). **b** wnt1 overexpression transfection promotes secretion of IL-6, TNF-α, and iNOS while *wnt1* siRNA transfection decreased them (*p* < 0.05). **c** *wnt1*siRNA transfection reversed p65 activation induced by LPS (*p* < 0.05). **d** *wnt1*siRNA transfection decreased secretion of IL-6, TNF-α, and iNOS induced by LPS (*p* < 0.05). Results were normalized against levels of GAPDH protein.

Levels of NF-kB protein were decreased in *wnt1* siRNA transfected THP-1 cells compared to negative control, while ectopic expression of wnt1 increased it (Fig. 2a). Transfection with wnt1 siRNA decreased the secretion of IL-6, TNF-α, and iNOS (Fig. 2b). Transfection with the wnt1 overexpression plasmid increased the secretion of IL-6, TNF-α, and iNOS (Fig. 2b). Furthermore, transfection with *wnt1* siRNA also decreased levels of NF-kB protein and the secretion of IL-6, TNF-α, and iNOS by LPS-stimulated THP-1 cells (Fig. 2c, d).

### Wnt1 Was Involved in LPS-Mediated NF-kB Activation Through Inducing SRA

**Fig. 3:**
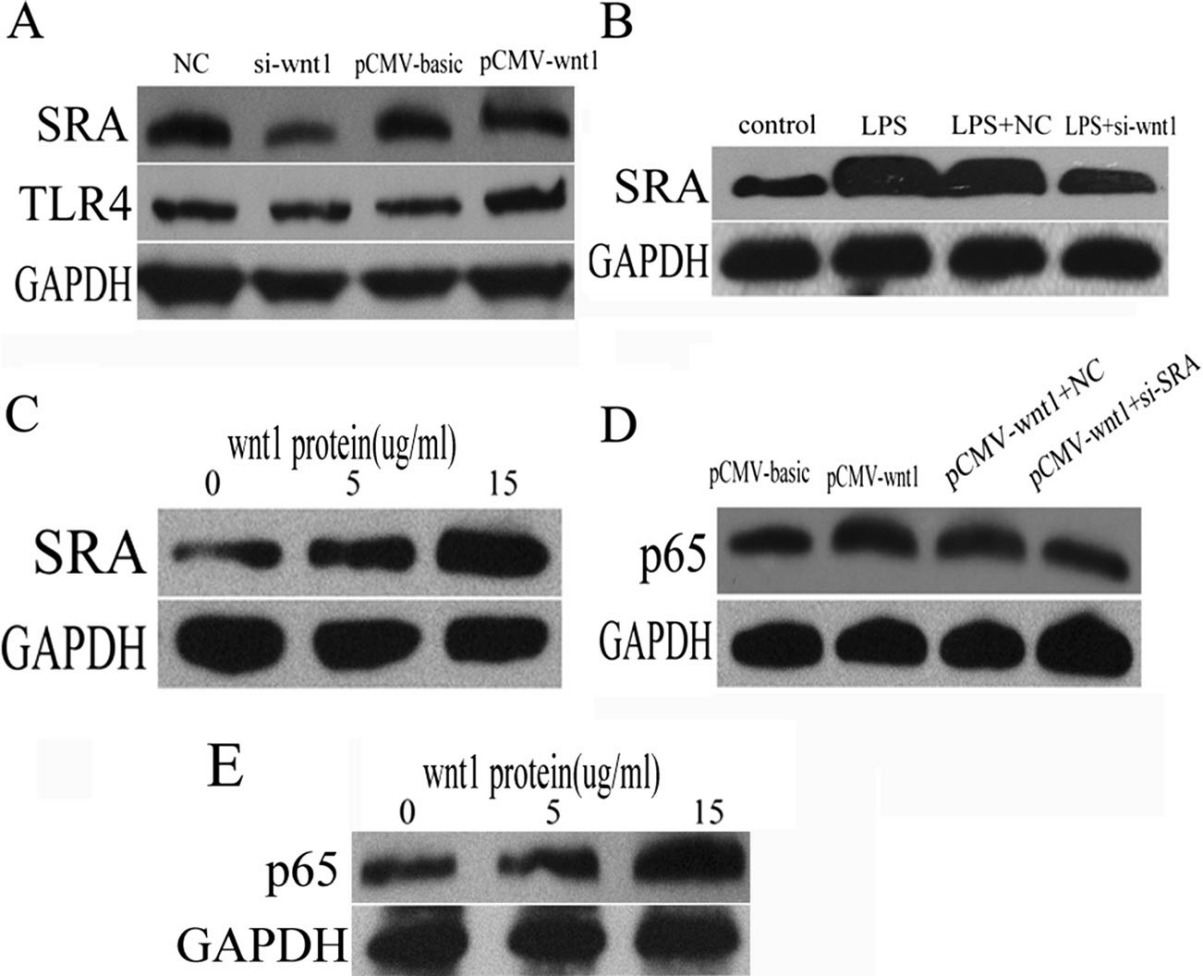
NF-kB was activated by wnt1 through increasing SRA expression. **a** SRA protein level was decreased in THP-1 cells transfected with wnt1 siRNA while ectopic expression of wnt1 increased it (*p* < 0.05), and TLR4 protein level was unchanged with either siRNA-wnt1 transfection or ectopic expression of wnt1. **b** *wnt1* siRNA transfection decreased SRA expression induced by LPS (*p* < 0.05). **c** SRA protein level was increased following concentration gradient of human wnt1 recombination protein (*p* < 0.05). **d** p65 protein level was decreased in wnt1 overexpression-transfected THP-1 cells treated with *SRA* siRNA (*p* < 0.05). **e** p65 protein level was increased following concentration gradient of human wnt1 recombination protein (from 0 to 15 ug/ml) (*p <* 0.05). Results were normalized against levels of GAPDH protein.

To explore the detail mechanism involved in NF-kB activation induced by wnt1, the expression of SRA andTLR4 was detected through transfection of *wnt1* siRNA or overexpression plasmid. As shown in Fig. 3a, wnt1 increased SRA expression but had no effect on levels of TLR4. Transfection with wnt1 siRNA also decreased levels of SRA protein by LPS-stimulated THP-1 cells (Fig. 3b). Moreover, levels of SRA protein were also increased in a dose-dependent manner of wnt1 recombination protein (Fig. 3c). Transfection with *SRA* siRNA decreased levels of NF-kB protein induced by *wnt1* overexpression (Fig. 3d), predicting that SRA was involved in wnt1-mediated NF-KB activation. More importantly, protein levels of p65 were also increased following concentration gradient of human wnt1 recombination protein (Fig. 3e).

### Wnt1 Induced SRA Expression Through Canonical wnt Pathway

**Fig. 4:**
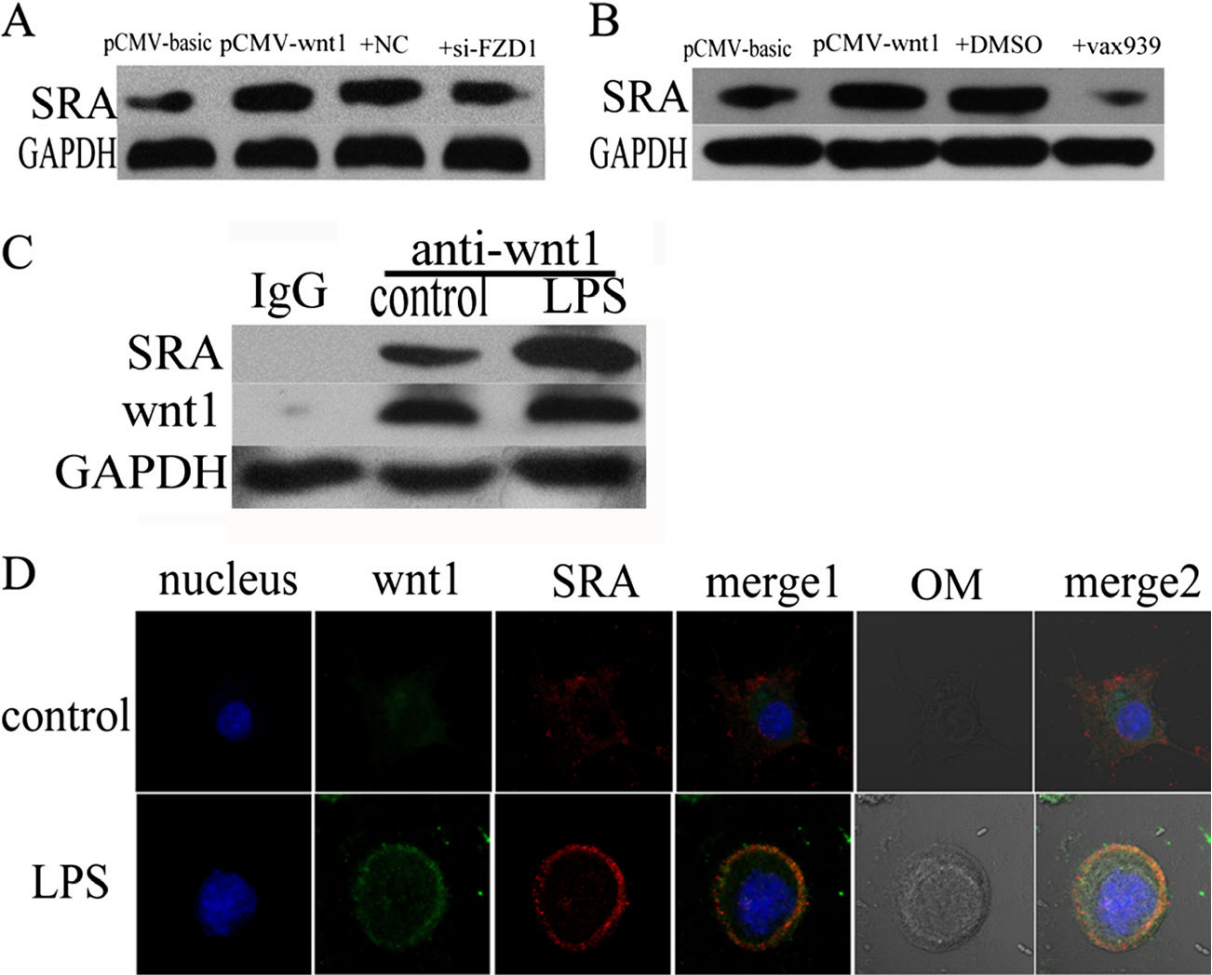
wnt1 promotes SRA expression through the canonical wnt pathway. **a–b** SRA protein level was decreased in wnt1 overexpression-transfected THP-1 cells treated with siRNA-FZD1 or VAX939 (β-catenin inhibitor) (*p* < 0.05). **c** Co-immunoprecipitation experiments showed an interaction between wnt1 and SRA (*p* < 0.05). LPS treatment strengthened it (*p* < 0.05). **d** THP-1 cells were cultured in the presence or absence of LPS (40 ug/ml) for 24 h. Confocal microscopy imaging of wnt1 (*green*), SRA (*red*), and optical microscopic view (*gray*) were shown. Results were normalized against levels of GAPDH protein.

The levels of SRA protein were decreased in LPS-stimulated THP-1 cells transfected with *FZD1* siRNA compared to negative control (Fig. 4a). The levels of SRA protein were also decreased in LPS-stimulated THP-1 cells treated with VAX939 (inhibitor of β-catenin) compared to untreated cells (Fig. 4b). Co-immunoprecipitation (IP) experiments were performed, and data showed that there was an interaction between wnt1 and SRA, and LPS stimulation strengthened it (Fig. 4c). Confocal image systems were also used to further assure there is the complex of wnt1 and SRA. More importantly, optical microscopy view show that the complex maybe localized in cell surface (Fig. 4d).

## DISCUSSION

This study demonstrates that wnt1-activated NF-kB mediates the inflammatory response by inducing SRA expression in the presence of LPS. The data indicate that the canonical wnt pathway participates in SRA activation induced by LPS. Furthermore, wnt1 is necessary for LPS-induction of SRA expression, and the canonical wnt pathway is responsible for SRA regulation by wnt1. These findings confirm that wnt1 plays an important role in mediating the transcription of multiple inflammatory factors in response to LPS stimulation. The results provide a new insight into the mechanism of LPS-induced NF-kB activation.

LPS released upon gram-negative bacteria infection and carried into the blood throughout the body results in a large-scale toxic and inflammatory response [9, 3]. The mechanism of NF-kB upregulation by LPS has been extensively researched. It is becoming clear that activation of TLR4 and SRA on the cell surface is involved in LPS-induced NF-kB activation.NF-kB activation and TLR4 mediate the secretion of LPS-induced inflammatory factors via a MyD88-dependent pathway [2]. And SRA may be involved in this process, particularly in the context of atherosclerosis [10]. Previous studies show that suppression of TLR4 or SRA reduced the LPS-induced inflammatory response in macrophages [11]. Activation of p38 and interaction of TLR4 and TLR2 agonists upregulated SRA expression in the presence of LPS [11]. TLRs can regulate phagocytosis mediated by SRA [12]. Furthermore, NF-kB increased the transient expression of wnt1 in PC12 cells [8]. But the relationship among wnt1, SRA, and NF-KB is unknown in macrophages. Moreover, whether wnt1 can regulate other molecules involved in inflammatory response induced by LPS was also ambiguous.

As an evolutionarily conserved transduction signaling, wnt1 participates in various biological activities including cellular transformation and cell proliferation through regulating the cell cycle [13], cyclooxygenase-2 [14], leptin [15], and matrix metalloproteinases [16]. The current study reveals that wnt1 also influences the secretion of LPS-induced inflammatory factors, including IL-6, TNF-α, and iNOS induced by LPS. To our best knowledge, this is the first report describing a role for wnt1 in the LPS-induced inflammatory factors. Further investigations are required to fully elucidate the mechanisms involved in this signaling cascade.

In brief, our study demonstrated that wnt1 promotes NF-KB activation induced by LPS through upregulating SRA. Furthermore, through inducing SRA, wnt1 can also mediate NF-kB activation and promote secretion of inflammatory factors including IL-6, TNFα, and iNOS. This discovery provided a new insight in inflammation caused by LPS and may be helpful in finding new therapy methods for endotoxima.
